# Supplementary material for: Fluorescence quenching in plasmonic dimers due to electron tunneling
Source: Nanophotonics. 2022 Jan 25;11(11):2473–82. doi: 10.1515/nanoph-2021-0707 (PMC11501688; doi:10.1515/nanoph-2021-0707)
Supplement: Supplementary file 1 — Supplementary Material Details [file j_nanoph-2021-0707_suppl.pdf]

Supplementary Material for
"Fluorescence quenching in plasmonic dimers due to electron tunneling"

Henrikh M. Baghramyan[1] and Cristian Ciracì[1*]
[1] *Center for Biomolecular Nanotechnologies, Istituto Italiano di Tecnologia, Via Barsanti 14, 73010 Arnesano (LE), Italy*
(Dated: December 23, 2021)

S1. THE EXCITATION ENHANCEMENT AND THE QUANTUM YIELD

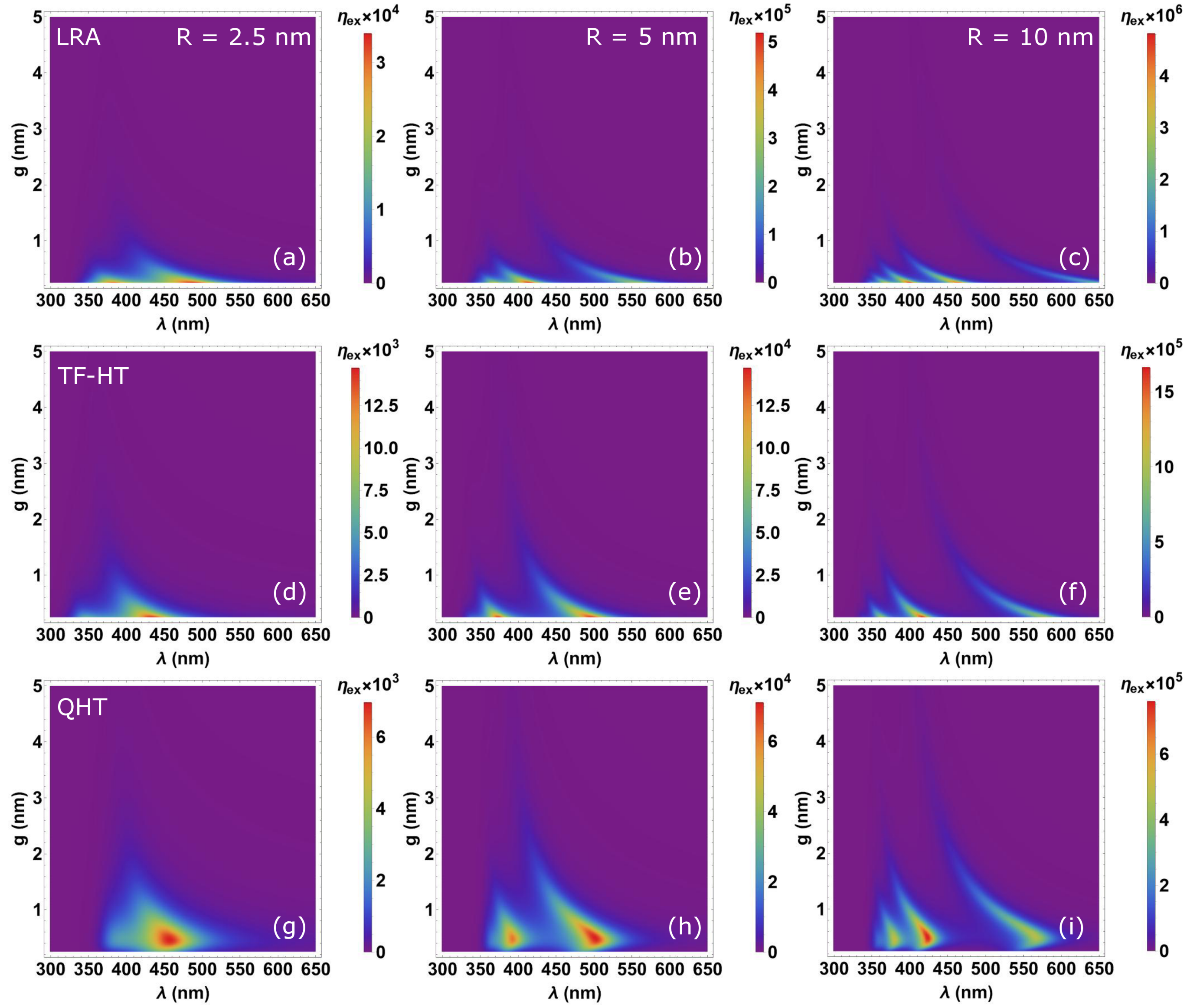

FIG. S1. The dependence of the excitation enhancement on the gap size g and the wavelength λ as computed from LRA, TF-HT and QHT for $R = 2.5; 5; 10$ nm.

* cristian.ciraci@iit.it

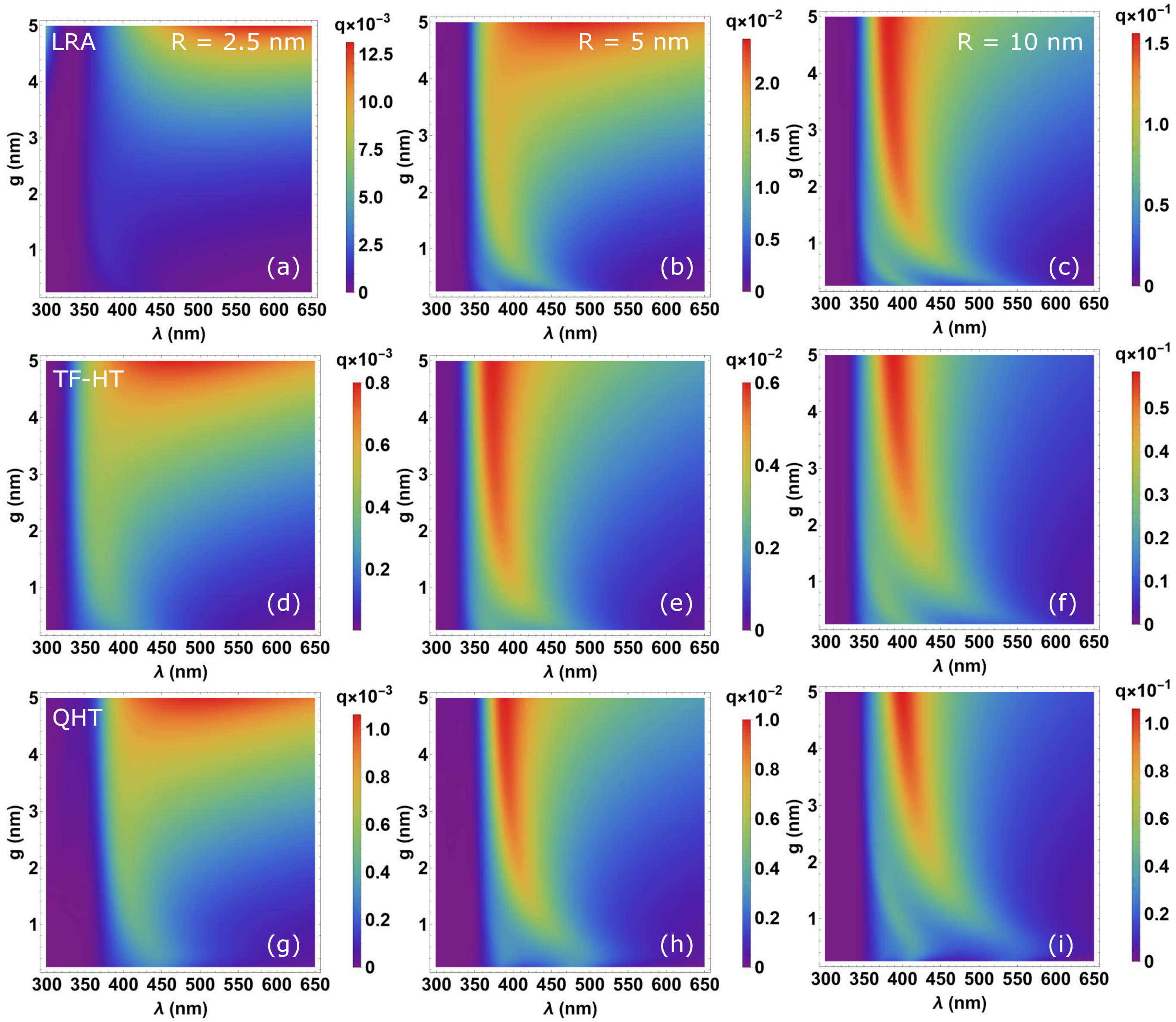

FIG. S2. The dependence of the quantum yield on the gap size g and the wavelength λ as computed from LRA, TF-HT and QHT for $R = 2.5; 5; 10$ nm.

S2. THE COMPUTATION DOMAIN AND THE MESHING.

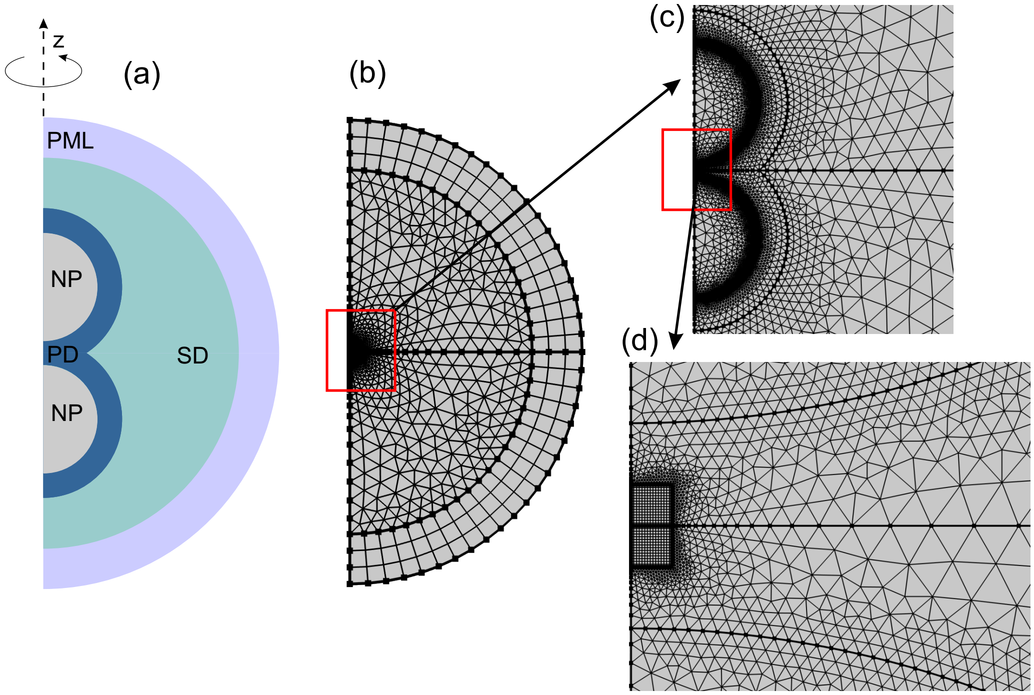

FIG. S3. The schematic images for the computation domain - (a), the meshing style used - (b) - (d).

S3. THE CURRENT DENSITY.

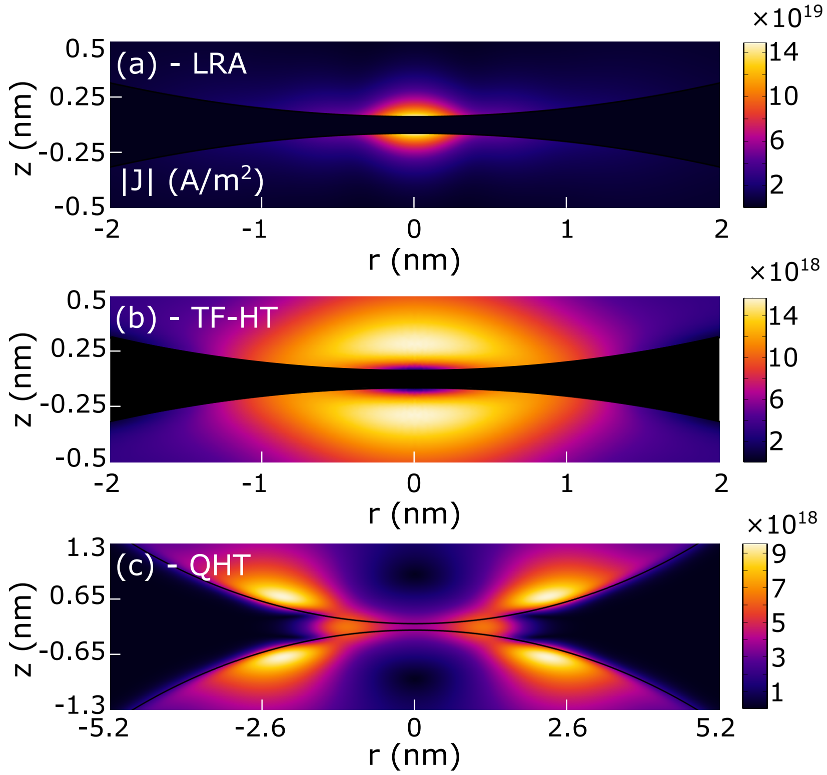

FIG. S4. Current density as obtained from LRA, TF and QHT at $\lambda = 400$ nm for $g = 0.1$ nm and $R = 10$ nm.

S4. THE POTENTIAL

The first-order terms in the potential

$$\left(\frac{\delta G\left[n\right]}{\delta n}\right)_1 = \left(\frac{\delta E_{\mathrm{XC}}^{\mathrm{LDA}}}{\delta n}\right)_1 + \left(\frac{\delta T_s}{\delta n}\right)_1 \tag{S1}$$

are presented below.

For the exchange-correlation potential, it is given by:

$$\left(\frac{\delta E_{\mathrm{XC}}^{\mathrm{LDA}}}{\delta n}\right)_1 = \left(E_h\right)\left(-a_0\frac{4}{9}c_X n_0^{-2/3} n_1 + a_0^3 \mu_C'\left[n_0\right] n_1\right), \tag{S2}$$

where

$$\mu_C'[n] = \begin{cases} -\frac{4\pi}{9}\left[a + \frac{1}{3}\left(1 + 2\ln\left(r_s\right)\right) c r_s + \frac{2}{3} d r_s\right] r_s^3, & r_s < 1, \\ \frac{\alpha\pi}{27}\frac{5\beta_1\sqrt{r_s} + \left(7\beta_1^2 + 8\beta_2\right) r_s + 21\beta_1\beta_2 r_s^{3/2} + 16\beta_2^2 r_s^2}{\left(1 + \beta_1\sqrt{r_s} + \beta_2 r_s\right)^3}, & r_s \geqslant 1, \end{cases} \tag{S3}$$

with $c_X = \frac{3}{4}\left(\frac{3}{\pi}\right)^{1/3}$ and the values of the coefficients are $a = 0.0311$, $b = -0.048$, $c = 0.002$, $d = -0.0116$, $\alpha = -0.1423$, $\beta_1 = 1.0529$, and $\beta_2 = 0.3334$.

The expression for $\left(\frac{\delta T_s}{\delta n}\right)_1$ is:

$$\left(\frac{\delta T_s}{\delta n}\right)_1 = \left(\frac{\delta T_s^{\mathrm{I}}}{\delta n}\right)_1 + \left(\frac{\delta T_s^{\mathrm{II}}}{\delta n}\right)_1 + \left(\frac{\delta T_s^{\mathrm{III}}}{\delta n}\right)_1, \tag{S4}$$

where

$$\left(\frac{\delta T_s^{\mathrm{I}}}{\delta n}\right)_1 = \tau_{nn}^{(0)} n_1 \tag{S5a}$$

$$\left(\frac{\delta T_s^{\mathrm{II}}}{\delta n}\right)_1 = -2\tau_{nnw}^{(0)}\left|\nabla n_0\right|^2 n_1 - 2\tau_{nw}^{(0)}\left[n_1\nabla^2 n_0 + \nabla n_0\cdot\nabla n_1\right] - 2\tau_w^{(0)}\nabla^2 n_1 \tag{S5b}$$

$$- 2\tau_{ww}^{(0)}\left[2\left(\nabla n_0\cdot\nabla n_1\right)\nabla^2 n_0 + \nabla\left(\left|\nabla n_0\right|^2\right)\cdot\nabla n_1 + 2\nabla n_0\cdot\nabla\left(\nabla n_0\cdot\nabla n_1\right)\right] \tag{S5c}$$

$$- 4\tau_{www}^{(0)}\left(\nabla n_0\cdot\nabla n_1\right)\left[\nabla n_0\cdot\nabla\left(\left|\nabla n_0\right|^2\right)\right] \tag{S5d}$$

$$- 2\tau_{nww}^{(0)}\left[2\left(\nabla n_0\cdot\nabla n_1\right)\left|\nabla n_0\right|^2 + \left\{\nabla n_0\cdot\nabla\left(\left|\nabla n_0\right|^2\right)\right\} n_1\right], \tag{S5e}$$

$$\left(\frac{\delta T_s^{\mathrm{III}}}{\delta n}\right)_1 = \tau_{nnnq}^{(0)}\left|\nabla n_0\right|^2 n_1 + \tau_{nnq}^{(0)}\left[2\nabla n_0\cdot\nabla n_1 + n_1\nabla^2 n_0\right] + 2\tau_{nq}^{(0)}\nabla^2 n_1 \tag{S5f}$$

$$+ \tau_{nnqq}^{(0)}\left[\left|\nabla n_0\right|^2\nabla^2 n_1 + 2n_1\left\{\nabla n_0\cdot\nabla\left(\nabla^2 n_0\right)\right\}\right] \tag{S5g}$$

$$+ \tau_{nqq}^{(0)}\left[\nabla^2 n_0\nabla^2 n_1 + 2\nabla n_1\cdot\nabla\left(\nabla^2 n_0\right) + 2\nabla n_0\cdot\nabla\left(\nabla^2 n_1\right) + \nabla^2\left(\nabla^2 n_0\right) n_1\right] \tag{S5h}$$

$$+ \tau_{qq}^{(0)}\nabla^2\left(\nabla^2 n_1\right) \tag{S5i}$$

$$+ \tau_{qqq}^{(0)}\left[2\left(\nabla\left(\nabla^2 n_0\right)\cdot\nabla\left(\nabla^2 n_1\right)\right) + \nabla^2\left(\nabla^2 n_0\right)\nabla^2 n_1\right] \tag{S5j}$$

$$+ \tau_{nqqq}^{(0)}\left[2\left\{\nabla n_0\cdot\nabla\left(\nabla^2 n_0\right)\right\}\nabla^2 n_1 + \left|\nabla\left(\nabla^2 n_0\right)\right|^2 n_1\right] \tag{S5k}$$

$$+ \tau_{qqqq}^{(0)}\left|\nabla\left(\nabla^2 n_0\right)\right|^2\nabla^2 n_1. \tag{S5l}$$

The subscripts $i = n, w, q$ denote the partial derivatives, and the superscript (0) means that the function is evaluated at $n = n_0$.

The potentials for each scenario considered in the article are as follows.

- For LRA there is no functional and hence $\left(\frac{\delta G[n]}{\delta n}\right)_1 = 0$.
- For TF-HT $\left(\frac{\delta G[n]}{\delta n}\right)_1$ is given by the Eq. (S5a).
- For QHT, Eqs. (S5a) and (S5b) are considered.
- For QHT-PGSL we use Eqs. (S5a)-(S5i).
- And finally, for QHT-PGSLN all the equations in (S5) have to be accounted for.
